# Predictors for Adherence to Treatment Strategies in Elderly HNSCC Patients

**DOI:** 10.3390/cancers14020423

**Published:** 2022-01-14

**Authors:** Raphaela Graessle, Carmen Stromberger, Max Heiland, Christian Doll, Veit M. Hofmann, Konrad Klinghammer, Ingeborg Tinhofer, Heidi Olze, Marcus Beck, Philipp Arens, Steffen Dommerich, Iris Piwonski, Annekatrin Coordes

**Affiliations:** 1 Department of Otorhinolaryngology, Head and Neck Surgery, Campus Virchow Klinikum and Campus Charité Mitte, Charité—Universitätsmedizin Berlin, Freie Universität Berlin, Humboldt-Universität zu Berlin, and Berlin Institute of Health, 10117 Berlin, Germany; raphaela.graessle@charite.de (R.G.); heidi.olze@charite.de (H.O.); philipp.arens@charite.de (P.A.); steffen.dommerich@charite.de (S.D.); 2 Department of Radiooncology, Charité—Universitätsmedizin Berlin, Freie Universität Berlin, Humboldt-Universität zu Berlin, and Berlin Institute of Health, 10117 Berlin, Germany; carmen.stromberger@charite.de (C.S.); ingeborg.tinhofer@charite.de (I.T.); marcus.beck@charite.de (M.B.); 3 Department of Oral and Maxillofacial Surgery, Campus Virchow Klinikum and Campus Benjamin Franklin, Charité—Universitätsmedizin Berlin, Freie Universität Berlin, Humboldt-Universität zu Berlin, and Berlin Institute of Health, 10117 Berlin, Germany; max.heiland@charite.de (M.H.); christian.doll@charite.de (C.D.); 4 Department of Otorhinolaryngology, Head and Neck Surgery, Campus Benjamin Franklin, Charité—Universitätsmedizin Berlin, Freie Universität Berlin, Humboldt-Universität zu Berlin, and Berlin Institute of Health, 10117 Berlin, Germany; veit.hofmann@charite.de; 5 Department of Hematology and Oncology, Campus Benjamin Franklin, Charité—Universitätsmedizin Berlin, Freie Universität Berlin, Humboldt-Universität zu Berlin, and Berlin Institute of Health, 10117 Berlin, Germany; konrad.klinghammer@charite.de; 6 Charité Comprehensive Cancer Center, 10117 Berlin, Germany; 7 Department of Pathology, Charité—Universitätsmedizin Berlin, Freie Universität Berlin, Humboldt-Universität zu Berlin, and Berlin Institute of Health, 10117 Berlin, Germany; iris.piwonski@charite.de

**Keywords:** head and neck squamous cell carcinoma, elderly patients, adherence, therapy, survival

## Abstract

**Simple Summary:**

The aim of this study was to find predictors for adherence to a therapy recommended by a multidisciplinary tumour board regarding 1125 elderly patients (70–100 years) with head and neck squamous cell carcinoma (HNSCC). The 5-year overall survival was significantly higher in adherent patients (45.1% versus 19.2%). Nonadherent patients were significantly more often smokers, drinkers, and had a worse tumour stage and lower health status (Karnofsky performance status). In contrast to the chronological patient age, the biological age (Charlson Comorbidity Index) was a significant predictor for adherence. The evaluated predictors for nonadherence need to be verified prospectively.

**Abstract:**

Finding a cure may be less important than ensuring the quality of life in elderly patients with head and neck squamous cell carcinoma (HNSCC). The aim of this study was to determine predictors for adherence. Clinical and pathological data from patients ≥70 years with HNSCC (initial diagnoses 2004–2018) were investigated retrospectively. Evaluated clinical predictors included biological age (Charlson Comorbidity Index; CCI), patient health (Karnofsky Performance Status; KPS) and therapy data. A total of 1125 patients were included. The median age was 75 years, 33.1% reached CCI ≥ 6, and 53.7% reached KPS ≤ 70%. In total, 968 patients were adherent, whereas 157 were nonadherent. Nonadherent patients were significantly more often smokers (*p =* 0.003), frequent drinkers (*p =* 0.001), had a worse health status (*p ≤* 0.001) and a lower biological age (*p =* 0.003), an advanced T classification and lymph node involvement or UICC stage (each *p ≤* 0.001). Approximately 88.0% of the included patients received a curative treatment recommendation. A total of 6.9% discontinued the therapy, and 7.0% refused the therapy. With the increasing complexity of a recommended therapy, adherence decreased. The 5-year overall survival was significantly higher in adherent patients (45.1% versus 19.2%). In contrast to the chronological patient age, biological age is a significant predictor for adherence. The evaluated predictors for nonadherence need to be verified prospectively.

## 1. Introduction

Life expectancy in the European Union has increased significantly from 77 in 2000 to 81 years in 2018 [1]. In Germany, life expectancy is 81 [2]. Therefore, the number of older patients with head and neck squamous cell carcinoma (HNSCC) has increased. Due to demographic change, by 2030, there may be an increase of 66% in laryngeal carcinomas and 61% in oral cavity or pharyngeal carcinomas in patients older than 65, compared to 2010 [3]. However, elderly patients are underrepresented in clinical trials. The proportion of patients ≥75 years generally represents less than 10% of study populations [4]. Due to this current data gap, a standard of care for this patient subgroup needs to be established [5]. Therapeutic recommendations are largely based on the medical assessment of the treating physicians and/or on clinic-specific guidelines. The need for standardised guidelines for the elderly is further supported by the fact that the older patients become, the more likely they are not to receive therapy according to the current standard. Older HNSCC patients have a significantly higher probability of being treated with palliative rather than curative therapy compared to younger patient groups [6,7,8].

Even though comorbidities are more common in geriatric patients and lead to a larger amount of non-standard treatment regardless of age, they are not the only explanation for this difference. Age itself seems to play a significant role. For instance, only 2% of patients between 45 and 60 without comorbidities do not receive a guideline-based therapy, whereas more than 10% of patients older than 70, also without comorbidities, are treated with non-standard therapies [7]. In this context, one must keep in mind that chronological age should not prevent patients from receiving curative treatment [9]. The comorbidities that increase the probability of treatment-related adverse events and predict a disadvantageous outcome are of higher importance [10,11,12,13].

Additionally, individual patients’ preferences are decisive in the choice of therapy. Being cured is not the most important objective for HNSCC patients in every case [14]. Older patients tend to put more emphasis on quality of life, and overall survival (OS) is less important to them [15].

In order to better understand the complex situation regarding the choice of treatment in older HNSCC patients, our primary objective was to determine predictors by which elderly patients decide whether to decline or discontinue therapies proposed by a multidisciplinary tumour board. Secondly, we attempted to find predictors for OS in elderly patients with HNSCC. Thirdly, we examined if there are OS predictors particular to adherent and nonadherent patients.

## 2. Materials and Methods

### 2.1. Patient Inclusion Criteria

The present study included HNSCC patients aged 70 years or older who were treated at the Charité-Universitätsmedizin Berlin between 2004 and 2018. In all cases, HNSCC was confirmed histologically. The following subsites of HNSCC were included: larynx, oro-/naso-/hypopharynx, oral cavity and nasal/paranasal sinuses. Clinicopathological data of all patients were documented and retrospectively extracted from electronic patient records. The study was approved by the local ethics committee (EA1/256/20).

### 2.2. Patient and Treatment Assessment

Each patient underwent common diagnostic procedures at our institution. This included a precise medical history and an examination of the head and neck, including endoscopy. Radiological investigations generally included a computed tomography (CT) scan or magnetic resonance imaging of the neck and a CT scan of the thorax and abdomen. Furthermore, patients underwent a panendoscopy to evaluate the tumour extension, exclude synchronic tumours and take tissue biopsies. Tissue samples of oropharyngeal squamous cell carcinoma were examined by immunohistochemistry to detect the expression of p16 as a surrogate marker for HPV association [16]. Since 2017, p16 was routinely stained. In previous cases, p16 status was investigated whenever sufficient samples were available. To compare patients adequately, the 8th edition of the UICC TNM classification, which takes account of p16 status, was used [17]. Oropharyngeal carcinomas for which no tissue sample was available for staining were therefore assumed to be p16 negative for purposes of TNM classification. For all other purposes, the p16 status of these cases was classified as “not applicable”. The treatment in all cases was planned based on a multidisciplinary head and neck tumour board recommendation (head and neck surgeons, medical and radiation oncologists, pathologists and radiologists). Patients who followed the board’s recommendation were considered to be adherent; the other patients (who discontinued or rejected the recommended therapy) were considered as nonadherent. The patients decided whether to follow the proposed therapy after an explanatory conversation with their attending physician, which addressed their needs and concerns. The ultimate decision of whether to follow the proposed therapy lay with the patient. Therapeutic recommendations by the tumour board were individual because comorbidities, especially with advanced age, sometimes limited the reasonable therapy options considerably. The medical condition of each patient prior to the therapy was taken into account using the Karnofsky performance status (KPS) [18]. For this study, comorbidities were scored retrospectively using the Charlson Comorbidity Index (CCI) based on the patients’ documented secondary diagnoses [19]. The CCI was used to represent the biological age of patients.

The surgical procedure aimed at an in-sano resection. A tracheostomy was used as a surgical procedure to secure the airway pathway. In tumours ≥T3 or histologically confirmed lymph node involvement, adjuvant radiotherapy/radiochemotherapy (RT/RCT) was indicated.

Adjuvant RT was usually performed using 54 to 66 Gy. In cases with high-risk features, 66 Gy and concomitant chemotherapy were used: in cases of resection with a close margin (<5 mm), or nodal extracapsular spread (ENE), tumour resection with microscopically detected tumour cells in the surgical margins (R1). Concomitant chemotherapy consisted of cisplatin (5 × 20 mg/m^2^, 1. and 5 weeks of RT, or weekly 30 mg/m^2^) ±5-fluorouracil (5FU, 5 × 600 mg/m^2^ c.i., 1st week of RT), or in the definitive setting, alternatively mitomycin C (2 × 10 mg/m^2^, d1 and d29)  ±5FU (5 × 600 mg/m^2^ c.i., 1st week of RT) or cetuximab (400 mg/m^2^ preload, and 250 mg/m^2^ weekly to RT).

Alternatively, definitive RT/RCT (>70 Gy) was performed (e.g., in the case of advanced nodal categories). Patients with stage I–II disease received definitive RT with 66–70 Gy according to international treatment guidelines (e.g., NCCN HN V1.2022). Patients with locally advanced HNSCC with multiple comorbidities, frailty and/or poor health who did not qualify for concurrent RCT had curative RT ≥70 Gy in altered fractionation (hyperfractionation) or normofractionation.

In some cases, including those with poor general health, palliative RT/RCT, systemic therapy (ST) including palliative chemotherapy or immunotherapy (e.g., cetuximab and nivolumab), or Best Supportive Care (BSC) were suggested by the tumour board.

### 2.3. Statstical Analysis

The data set was analysed using IBM SPSS Statistics version 26.0.0.0 for macOS (IBM Corp., Armonk, NY, USA).

Patients’ characteristics were reported according to the sample Guidelines [20]. Data not normally distributed, (pack years and age at initial diagnosis) was summarised with medians and ranges with minimum and maximum values. For statical processing, several variables were converted into dichotomous values. In this study, an exploratory data analysis was performed, and all *p*-values were reported without adjustment for multiple testing.

The primary objective was to identify constitutional differences in adherent and nonadherent patients. The chi-square test was used to test the categorical variables of characteristics among adherent and nonadherent patients for significant differences. The Mann–Whitney U test was used with the same intention for the metric, non-normally distributed variables. The following clinicopathological variables were recorded: sex (male vs. female), age at initial diagnosis of HNSCC, tobacco exposure (non-smoker vs. former/current smoker), pack years, alcohol abuse (no ethanol consumption vs. ethanol consumption), additional cancer diagnoses (other cancers vs. none), number of additional cancer diagnoses (0 vs. 1 vs. ≥2), CCI (≤5 vs. ≥6), KPS (≤70% vs. ≥80%), death due to cancer (survived vs. non-cancer-associated vs. cancer-associated), tumour site (oropharynx vs. oral cavity vs. larynx vs. hypopharynx vs. nasal/paranasal sinus vs. nasopharynx), p16 in oropharynx carcinomas only (positive vs. negative), tumour grading (G1 vs. G2 vs. G3), T classification (T1–2 vs. T3–4), N classification (positive vs. negative), M classification (positive vs. negative), UICC stage (I–II vs. III–IV), received treatment (BSC vs. pall. RT/RCT vs. surgery vs. surgery + adj. RT/RCT vs. def. RT/RCT vs. ST), recommended treatment (BSC vs. pall. RT/RCT vs. surgery vs. surgery + adj. RT/RCT vs. def. RT/RCT vs. ST), intention of therapy (curative vs. palliative vs. curative, discontinued) and implementation of therapy (discontinued vs. rejected vs. carried out).

Secondly, OS and disease-free survival (DFS) of the patient cohort was analysed using the Kaplan–Meier method. We proceeded to a univariant analysis to identify variables that significantly influence OS. For this purpose, the log-rank test was used. The OS was defined as the time between the initial diagnosis of the HNSCC and the date of death or last follow-up. Almost the same variables were included; only pack years, the number of additional cancer diagnoses and death due to cancer were not analysed. DFS was defined as the time between the initial diagnosis of the HNSCC and the time of recurrence, death or last follow-up. The log-rank test was used to analyse the influence of adherence on DFS.

For multivariate analyses of OS, the Cox proportional hazards model was used. The following variables were considered: age at initial diagnosis of HNSCC, tobacco exposure, CCI, KPS, UICC stage and adherence to treatment recommendation.

For all tests, *p*-values < 0.05 were assumed to be statistically significant.

## 3. Results

### 3.1. Patient Characteristics

During the study period, 1353 patients aged 70 years or older were diagnosed with HNSCC at the Charité-Universitätsmedizin Berlin. The present study included 1125 of these patients. In all patients, a complete tumour stage, a recommendation by the multidisciplinary tumour board on treatment and the course of therapy were documented. In the remaining 228 patients, the data was incomplete. The clinicopathological data is outlined in Table 1.

The study population contained chiefly male patients (*n* = 759, 67.5%); the median age was 75 and ranged from 70 to 100. In 641 patients (57.0%), smoking status could be investigated; 65.5% (*n* = 420) of patients were current or former smokers. In 262 patients, the number of pack years (PY) with a median of 50 PY (range: 3–200 PY) was recorded. Alcohol consumption was documented for 633 patients, and 199 (31.4%) patients regularly consumed alcohol. Approximately 33.1% (*n* = 372) had a history of cancer other than HNSCC.

The study population had a mixed health status: 33.1% of the cases reached a CCI ≥ 6, whereas 46.3% of patients achieved more than 80% on the KPS. A total of 627 (55.7%) patients died during the follow-up period of up to 170 months (range: 0–170; median 23 months), 67.9% (*n* = 426) of these deaths were known to be related to the HNSCC diagnosis.

Tumours affected the oropharynx (*n* = 305, 27.1%), oral cavity (*n* = 449, 39.9%), larynx (*n* = 215, 19.1 %), hypopharynx (*n* = 95, 8.4%), nasal/paranasal sinuses (*n* = 43, 3.8%) and nasopharynx (*n* = 18, 1.6%). Predominantly, the patients’ tumour stage was advanced (59.9% UICC III-IV) at the time of the initial diagnosis. In total, 47.9% (*n* = 539) of the study population were classified T3-4 and in 48.3% of cases (*n* = 543) regional lymph nodes were affected by the HNSCC. Distant metastases were found in 44 patients (3.9%) at the time of diagnosis of HNSCC.

All tumours were confirmed histologically as being of squamous cell origin, and were graded as G1 (*n* = 105, 10.3%), G2 (*n* = 657, 64.7%) or G3 (*n* = 253, 24.9%). In 59.7% of oropharyngeal carcinomas (*n* = 182), the p16-status was available. It was positive in 51.1% of cases (*n* = 93).

**Figure 1:**
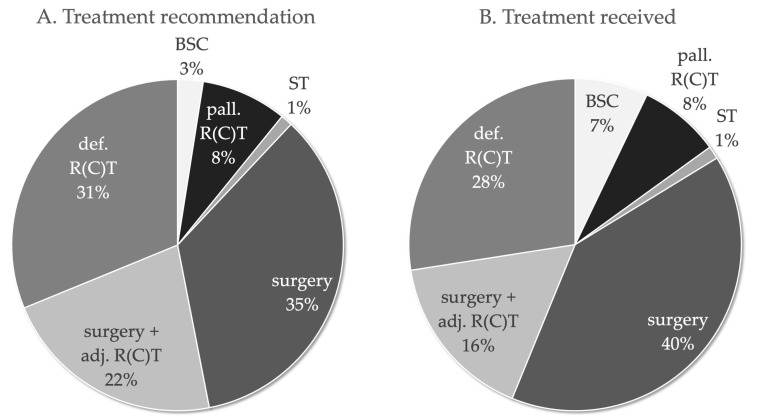
Treatment recommendations of the multidisciplinary tumour board of 1125 patients with HNSCC ≥ 70 years. (**A**). Treatment recommended and (**B**). treatment received.

The treatment recommendation of the multidisciplinary tumour board included (Figure 1): surgery (*n* = 393, 34.9%), definitive RT/RCT (*n* = 351, 31.2%), surgery with adjuvant RT/RCT (*n* = 246, 21.9%), palliative RT/RCT (*n* = 94, 8.4%), BSC (*n* = 28, 2.5%) and ST (*n* = 13, 1.2%). The tumour board recommended a curative therapy in 88.0% (*n* = 990) of cases. In 6.9% (78/1125) of cases, recommended therapy was discontinued (*n* = 78) and in 7.0% of cases the recommended therapy was rejected (*n* = 79). The treatments received included: surgery (*n* = 449, 39.9%), definitive RT/RCT (*n* = 309, 27.5%), surgery with adjuvant RT/RCT (*n* = 184, 16.4%), palliative RT/RCT (*n* = 89, 7.9%), BSC (*n* = 80, 7.1%) and ST (*n* = 14, 1.2%). The intention of the treatments that were actually implemented was also curative in most cases (*n* = 860, 76.4%).

Table 2 show a cross-table comparing the therapy recommended by the tumour board to the therapy actually received. Eighty-two patients (7.3%) originally started with a curative therapy but did not follow through until completion. Approximately 16.3% of the study population received treatment with palliative intent (*n* = 183). The median total dose of curative and palliative radiation was 70.0 and 45.0 Gy, respectively (45.0–75.6 Gy and 15.0–60.0 Gy).

### 3.2. Predictors for Nonadherence

According to their adherence to the tumour board recommendation, patients were divided into adherent (*n* = 968, 86.0%) and nonadherent (*n* = 157, 14.0%) groups. The TNM classification and UICC stage were significantly more advanced in the nonadherent subgroup (T *p ≤* 0.001; N *p ≤* 0.001; UICC *p ≤* 0.001). In total, 81.5% (*n* = 128) of nonadherent patients were diagnosed with a UICC stage III–IV compared to 56.4% (*n* = 546) of adherent patients. Nonadherent patients died significantly more often in association with their tumour than adherent patients (70.4% versus 37.3%; *p ≤* 0.001). Significant differences in the constitution of the groups were found in tobacco exposure (*p =* 0.003) and alcohol abuse (*p =* 0.001). Approximately 63.3% (*n* = 350) of all adherent patients were smokers, in contrast to 79.5% (*n* = 70) of nonadherent patients. Of all the adherent patients, 29.0% (*n* = 160) consumed larger quantities of alcohol, in contrast to 47.6% (*n* = 39) of nonadherent patients. There was also a difference in the patients’ health status (KPS *p ≤* 0.001). A total of 49.8% (*n* = 482) of the adherent subgroup versus 77.7% (*n* = 122) of the nonadherent group did not achieve more than 80% on the KPS. Biological age was significantly lower in nonadherent patients (CCI *p =* 0.003), while chronological age did not have any influence. A CCI of 6 or more was present in 31.4% (*n* = 304) of cases in the adherent subgroup versus 43.3% (*n* = 68) in the nonadherent subgroup.

**Figure 2:**
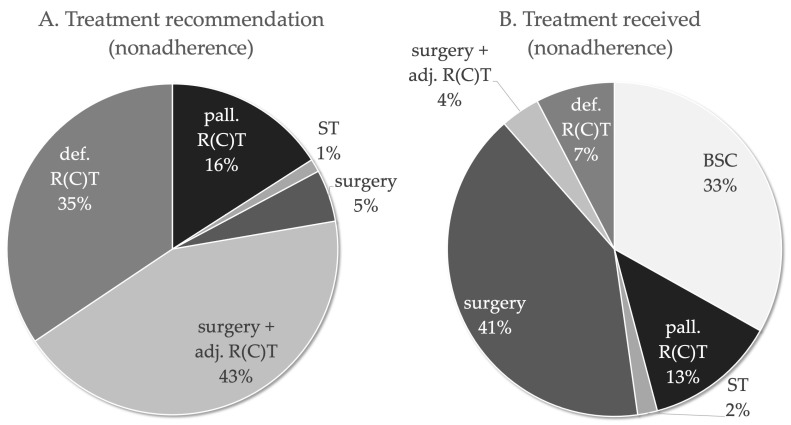
Treatment recommendations of the multidisciplinary tumour board of the nonadherent HNSCC patients ≥ 70 years. (**A**). Treatment recommended and (**B**). Treatment received by nonadherent patients.

In the adherent group, the therapy performed was, by definition, identical to the recommended therapy. The recommended curative treatment in nonadherent patients (Figure 2) was surgery with adjuvant RT/RCT in 43.3% of cases, surgery alone in 5.1% and definitive RT/RCT in 34.4%. By contrast, 3.8% of nonadherent patients actually received surgery with adjuvant RT/RCT, 40.8% received surgery only and 7.6% received definitive RT/RCT.

There were no cases (0.0%) in which the recommended palliative treatment in nonadherent patients was BSC, whereas it was palliative RT/RCT in 15.9% of cases and ST in 1.3%. By contrast, 33.1% of nonadherent patients actually received BSC, 12.7% of nonadherent patients received palliative RT/RCT and 1.9% of nonadherent patients received ST. Table 3 show a cross-table comparing the therapy recommended by the tumour board with the implementation of the therapy. In total, 28.0% (*n* = 44) of the nonadherent patients rejected adjuvant RT/RCT after surgery, and 23.6% (*n* = 37) of the nonadherent patients interrupted definitive RT/RCT. Three patients refused to complete surgery, i.e., opted not to undergo a recommended re-resection in R1 condition. Approximately 8.9% (*n* = 14) of nonadherent patients discontinued palliative RT/RCT. Forty-eight patients with a curative treatment recommendation according to tumour board opted for a palliative approach including ST, RT/RCT or BSC.

As previously mentioned, in 88.0% (*n* = 990) of cases, the treatment originally recommended by the tumour board was curative. The clinicopathological data of this subgroup are summarised in Supplementary Tables S1–S3. Here, differences between adherent and nonadherent patients were found for the same characteristics as in the whole study population.

### 3.3. Long-Term Survival and Disease Free Survival

**Figure 3:**
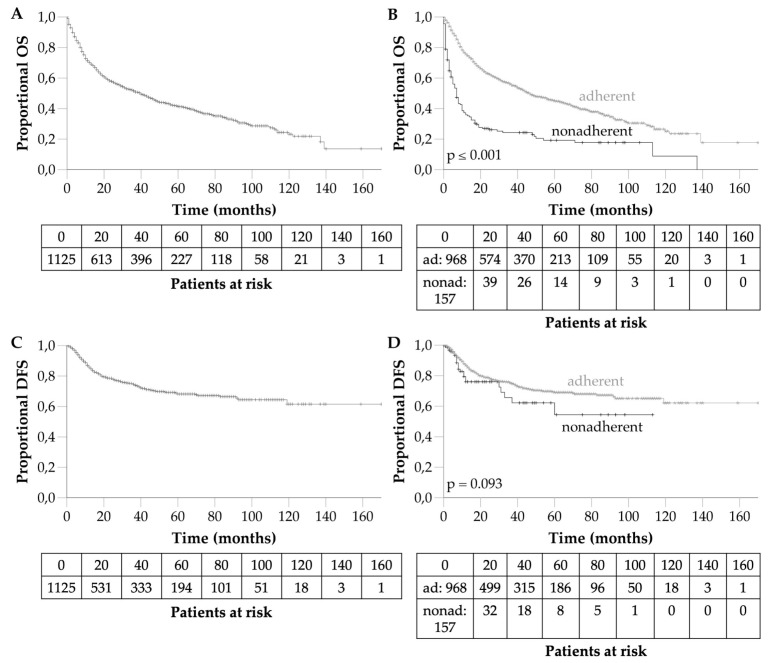
(**A**). Overall survival of the 1125 patients with HNSCC ≥ 70 years. (**B**). Overall survival depending on adherence. (**C**). Disease-free survival of the 1125 patients with HNSCC ≥ 70 years. (**D**). Disease-free survival depending on adherence. Ad, adherent; nonad, nonadherent.

The mean survival was 63 months (95%CI 57.98–68.75). The 1-, 3- and 5-year OS rates were 70.2, 51.6 and 41.5%, respectively (Figure 3A). Half of all patients (*n* = 627, 55.7%) died during the follow-up period, and in 67.9%, the patient’s death was known to be cancer-related. In 77.5% of cancer-associated deaths (*n* = 330), the tumour stage was already advanced (UICC III–IV) at initial diagnosis. The mean DFS was 118 months (95%CI 111.09–124.05). The 1-, 3- and 5-year DFS rates were 86.0, 74.6 and 68.2%, respectively (Figure 3C).

### 3.4. Predictors for Survival

Significant predictors for survival are summarised in Table 4. They included both chronological age (*p ≤* 0.001) and biological age (CCI, *p ≤* 0.001), tobacco exposure (*p =* 0.001), alcohol abuse (*p ≤* 0.001), good health (CCI ≤ 5, *p ≤* 0.001 and KPS ≥ 80%, *p ≤* 0.001), T classification (*p ≤* 0.001), lymph node involvement (*p ≤* 0.001), distant metastases (*p ≤* 0.001) and UICC stage (*p ≤* 0.001). Long-term survival in nonadherent patients was significantly worse compared to adherent patients (*p ≤* 0.001, Figure 3B). The 5-year OS was 45.1% for adherent patients compared to nonadherent patients (19.2%). The DFS in nonadherent patients tended to be worse compared to adherent patients (*p =* 0.093, Figure 3D). The multivariate analysis confirmed the independent influence on OS of chronological age, tobacco exposure, biological age, KPS, UICC stage and adherence to treatment recommendation.

In adherent patients, the predictors were mostly identical compared to the whole patient group (Table 4). In nonadherent patients, unlike in adherent patients, age, tobacco exposure, T classification, distal metastases, UICC stage and tumour site did not have any significant impact.

## 4. Discussion

This study attempted to find predictors for nonadherence to a recommended treatment regimen in elderly patients with HNSCC. The study included 1125 patients older than 70 (and up to 100). The 5-year OS was 41.5%. Predictors for survival included age, tobacco exposure, alcohol abuse, good health, T classification, lymph node involvement, distant metastases, UICC stage and adherence to the recommended treatment. These factors are comparable with the literature [21,22,23,24,25,26,27,28].

Since the definition of “elderly patients” with HNSCC varies in the literature, we decided to set our threshold age at ≥70. Even though this age limit is commonly used in other studies [29,30,31], there are also different interpretations. Some authors have used a lower (60 and 65 years) [32,33] or higher (80 years) [34,35] minimum age.

We also found some studies on the question of what distinguishes patients who were treated according to the guideline and were adherent from those who were nonadherent [6,7,22,36,37]. The studies used different guidelines as a basis for their research. Derks et al. [7] and Dronkers et al. [6] used the national guidelines published by the Comprehensive Cancer Centre the Netherlands (IKNL), Sanabria et al. [22] used hospital guidelines and Kusaba et al. [36] did not elaborate on the term standard therapy and referred to it as curative recommended therapy. Our approach is most consistent with the definition of Roden et al. [37], in which the recommended therapy is defined as standard therapy in accordance with the National Comprehensive Cancer Network (NCCN) guidelines, including patient-specific treatment [38].

In accordance with previous studies, we showed that patients who followed the treatment advice of the multidisciplinary tumour board benefited from a higher OS than patients who discontinued or declined a proposed therapy [6,22,36]. In this context, our results differed significantly from those of Roden et al. [37]. In their study, no significant difference in OS was found between the two patient groups. However, in this study, patients who refused therapy were grouped in the nonadherent group together with those who discontinued therapy. The reasons for discontinuation of therapy were not systematically recorded, but often it was due to deterioration of patients’ general condition that did not allow continuation of therapy and was often accompanied by earlier deaths.

Positive smoking status significantly reduces OS in HNSCC patients [23,24,25]. According to our research, however, this finding does not apply to the group of nonadherent patients. It appears that when patients deviate from their recommended therapy, smoking status no longer has an impact on OS. The same applies to alcohol abuse.

The tumour stage was a predictive factor for declining or discontinuing a recommended treatment [6,7,22]. With the increasing complexity of the recommended therapy, the adherence to the recommendation decreased. Approximately 28.0% of the nonadherent patients denied adjuvant RT/RCT after surgery, and 23.6% of the nonadherent patients interrupted definitive RT/RCT. The reason for this could be the longer duration of therapy if definitive RT/RCT or surgery with adjuvant RT/RCT are applied. In addition, the fear of side effects, especially from radiation and chemotherapy, could play a role. Furthermore, patients could be satisfied with their putative cancer-free condition after surgery without taking into consideration the importance of adjuvant therapy. Forty-eight patients with curative treatment recommended according to the tumour board received palliative care including ST, RT/RCT or BSC.

In elderly patients, there is a high prevalence of comorbidities. Advanced comorbidity has been shown to cause a marked reduction in life span in patients with HNSCC [26,27,28], as was corroborated by this study. Comorbidities cause an increased number of severe complications regardless of the therapy type [27,39]. It was previously shown that both KPS and CCI could serve as predictors for OS independently from one another [40]. This is supported by our multivariate analysis. Comorbidities are also an important prognostic factor in elderly patients receiving a standard curative treatment [6,7,22,41].

In this study, biological age and patient health were significant predictors for adherence in contrast to chronological age. However, it is difficult to compare the different studies in detail, as no uniform index was used to evaluate the comorbidities. For example, the Kaplan–Feinstein Index or the Adult Comorbidity Evaluation-27 (ACE-27) were used [6,7,22,28,42]. The CCI was used by Roden et al. [37], and in contrast to our current study, they could not identify any difference between both groups. One reason may be that considerably more patients were included (1125 vs. 159 patients) in our studies. This study confirms that the health status determined by KPS is an important factor for nonadherence [7,22].

There are other studies that partly address our objective and take greater account of the social status of the patients. For example, some studies suggest that marital status has an important impact on patients’ treatment (not limited to but including HNSCC patients) [6,7,43]. The lack of information on patients’ social networks may be the main drawback of this study. Another interesting aspect is the patients’ attitude to life. Even though being cured is the highest priority among patients in all age groups, it is quite conceivable that older patients, who usually make up a smaller proportion of the study participants, have different priorities and value quality of life to a higher degree [15,44]. Future research remains desirable due to the purely retrospective character of this study.

## 5. Conclusions

This study gives a comprehensive overview of clinicopathological data on elderly patients suffering from HNSCC treated at a large head and neck tumour centre in Germany. In contrast to chronological patient age, biological age was identified as a significant predictor for adherence. Further predictors for nonadherence include smoking, alcohol abuse, health status, tumour stage and complex therapy such as surgery with adjuvant RT/RCT. These findings need to be verified by prospective study designs.

## Figures and Tables

**Table 1:** Patient and tumour characteristics of the study population according to adherence/nonadherence to the tumour board recommendation.

Variable	Total	Adherence	Nonadherence	*p*-Value
	*n* = 1125	*n* = 968	*n* = 157	
Sex—no. (%)				0.572
Male	759 (67.5)	650 (67.1)	109 (69.4)	
Female	366 (32.5)	318 (32.9)	48 (30.6)	
Age at initial diagnosis of HNSCC, years				0.810
Median (range)	75 (30)	75 (30)	75 (24)	
Tobacco exposure—no. (%)				0.003
Non-smoker	221 (34.5)	203 (36.7)	18 (20.5)	
Current/former smoker	420 (65.5)	350 (63.3)	70 (79.5)	
Pack years				0.013
Median (range)	50 (197)	50 (147)	50 (195)	
Alcohol abuse—no. (%)				0.001
No ethanol consumption	434 (68.6)	391 (71.0)	43 (52.4)	
Ethanol consumption	199 (31.4)	160 (29.0)	39 (47.6)	
Additional cancer diagnoses—no. (%)				0.369
Other cancers	372 (33.1)	325 (33.6)	47 (29.9)	
None	753 (66.9)	643 (66.4)	110 (70.1)	
Number of additional cancer diagnoses				0.642
0	753 (66.9)	643 (66.4)	110 (70.1)	
1	291 (25.9)	255 (26.3)	36 (22.9)	
≥2	81 (7.2)	70 (7.2)	11 (7.0)	
Charlson Comorbidity Index—no. (%)				0.003
≤5	753 (66.9)	664 (68.6)	89 (56.7)	
≥6	372 (33.1)	304 (31.4)	68 (43.3)	
Karnofsky Performance Status—no. (%)				≤0.001
≤70%	604 (53.7)	482 (49.8)	122 (77.7)	
≥80%	521 (46.3)	486 (50.2)	35 (22.3)	
Death due to cancer				≤0.001
Survived	496 (48.8)	461 (52.7)	35 (24.6)	
Non-cancer-associated	94 (9.3)	87 (10.0)	7 (4.9)	
cancer-associated	426 (41.9)	326 (37.3)	100 (70.4)	
HNSCC characteristics				
Site of primary tumour—no. (%)				0.335 ^1^
Oropharynx	305 (27.1)	252 (26.0)	53 (33.8)	
Oral cavity	449 (39.9)	393 (40.6)	56 (35.7)	
Larynx	215 (19.1)	186 (19.2)	29 (18.5)	
Hypopharynx	95 (8.4)	81 (8.4)	14 (8.9)	
Nasal/paranasal sinuses	43 (3.8)	39 (4.0)	4 (2.5)	
Nasopharynx	18 (1.6)	17 (1.8)	1 (0.6)	
P16 in Oropharynx-Carcinoma—no. (%)				0.521
Positive	93 (51.1)	75 (50.0)	18 (56.3)	
Grading—no. (%)				0.002
G1	105 (10.3)	99 (11.4)	6 (4.7)	
G2	657 (64.7)	569 (65.3)	88 (61.5)	
G3	253 (24.9)	204 (23.5)	49 (34.3)	
T classification (T)—no. (%)				≤0.001
T1–2	586 (52.1)	536 (55.4)	50 (31.8)	
T3–4	539 (47.9)	432 (44.6)	107 (68.2)	
N classification—no. (%)				≤0.001
Positive	543 (48.3)	445 (46.0)	98 (62.4)	
M classification—no. (%)				0.087
Positive	44 (3.9)	34 (3.5)	10 (6.4)	
UICC stage (8th edition)—no. (%)				≤0.001
I–II	451 (40.1)	422 (43.6)	29 (18.5)	
III–IV	674 (59.9)	546 (56.4)	128 (81.5)	
Intention of therapy				≤0.001
Curative	860 (76.4)	860 (88.8)	-	
Palliative	183 (16.3)	108 (11.2)	77 (47.8)	
Curative, discontinued	82 (7.3)	-	82 (52.2)	

HNSCC, head and neck squamous cell carcinoma; UICC, Union for International Cancer Control. ^1^ The requirements to perform a chi-square test were not fulfilled.

**Table 2:** Cross-table comparing the recommended therapy to the received therapy.

		Treatment Received						Total
		BSC	Pall. R(C)T	ST	Surgery	Surgery + adj. R(C)T	Def. R(C)T	
Treatment recommendation	BSC	28 (2.5%)	0 (0.0%)	0 (0.0%)	0 (0.0%)	0 (0.0%)	0 (0.0%)	28 (2.5%)
	Pall. R(C)T	18 (1.6%)	74 (6.6%)	2 (0.2%)	0 (0.0%)	0 (0.0%)	0 (0.0%)	94 (8.4%)
	ST	2 (0.2%)	0 (0.0%)	11 (1.0%)	0 (0.0%)	0 (0.0%)	0 (0.0%)	13 (1.2%)
	Surgery	4 (0.4%)	1 (0.1%)	0 (0.0%)	388 (34.5%)	0 (0.0%)	0 (0.0%)	393 (34.9%)
	Surgery + adj. R(C)T	0 (0.0%)	1 (0.1%)	0 (0.0%)	61 (5.4%)	184 (16.4%)	0 (0.0%)	246 (21.9%)
	Def. R(C)T	28 (2.5%)	13 (1.2%)	1 (0.1%)	0 (0.0%)	0 (0.0%)	309 (27.5%)	351 (31.2%)
Total		82 (7.3%)	89 (7.9%)	14 (1.2%)	447 (39.7%)	184 (16.4%)	309 (27.5%)	1125 (100%)

BSC, Best Supportive Care; Pall. R(C)T, palliative radio(chemo)therapy; ST, systemic therapy; Adj. R(C)T, adjuvant radio(chemo)therapy; Def. R(C)T, definitive radio(chemo)therapy.

**Table 3:** Cross-table comparing the therapy recommended by the tumour board to the implementation of the therapy.

		Nonadherent		Adherent	Total
		Discontinued	Rejected	Carried out	
Treatment recommendation	Palliative/ BSC	0 (0.0%)	0 (0.0%)	28 (2.5%)	28 (2.5%)
	Pall. R(C)T	14 (1.2%)	11 (1.0%)	69 (6.1%)	94 (8.4%)
	ST	0 (0.0%)	2 (0.2%)	11 (1.0%)	13 (1.2%)
	Surgery	3 (0.3%)	5 (0.4%)	385 (34.2%)	393 (34.9%)
	Surgery + adj. R(C)T	24 (2.1%)	44 (3.9%)	178 (15.8%)	246 (21.9%)
	Def. R(C)T	37 (3.3%)	17 (1.5%)	297 (26.4%)	351 (31.2%)
Total		78 (6.9%)	79 (7.0%)	968 (86.0%)	1125 (100%)

BSC, Best Supportive Care; Pall. R(C)T, palliative radio(chemo)therapy; ST, systemic therapy; Adj. R(C)T, adjuvant radio(chemo)therapy; Def. R(C)T, definitive radio(chemo)therapy.

**Table 4:** Univariate and multivariate analysis of clinicopathologic variables associated with overall survival (Table S4).

Univariate Analysis										
Variable		Total			Adherence			Nonadherence		
		*n* = 1125	Mean OS (Months/% ^1^)	*p* Value ^2^	*n* = 968	Mean OS (Months/% ^1^)	*p* Value ^2^	*n* = 157	Mean OS (Months/% ^1^)	*p* Value ^2^
Sex				0.664			0.779			0.460
	Male	759	64/41.1		650	69/44.7		109	27/19.5	
	Female	366	60/42.2		318	64/45.8		48	33/19.0	
Age at initial diagnosis of HNSCC				≤0.001			≤0.001			0.062
	70–74 years	506	70/45.9		432	77/50.3		74	33/20.2	
	75–79 years	388	56/40.1		342	61/43.1		46	21/18.5	
	80–84 years	143	51/34.1		114	52/36.3		29	35/24.8	
	85–89 years	61	41/36.2		57	44/38.8		4	4/0.0	
	Older than 90 years	27	20/23.1		23	24/27.8		4	4/0.0	
Tobacco exposure				0.001			0.003			0.664
	Non-smoker	221	70/52.2		203	74/54.7		18	25/27.8	
	Current/former smoker	420	55/35.5		350	60/39.3		70	26/15.7	
Alcohol abuse				≤0.001			≤0.001			0.021
	No ethanol consumption	434	69/52.5		391	46/55.5		43	16/8.2	
	Ethanol consumption	199	40/23.1		160	72/26.8		39	37/22.9	
Additional cancer diagnoses				0.060			0.073			0.080
	Other cancers	372	54/37.1		325	58/40.5		47	19/13.3	
	None	753	67/43.8		643	75/47.6		110	35/21.7	
Charlson comorbidity index				≤0.001			≤0.001			0.025
	≤5	753	74/48.5		644	80/51.5		89	39/25.7	
	≥6	372	39/27.1		304	44/30.6		68	17/11.5	
Karnofsky performance status				≤0.001			≤0.001			0.004
	≤70%	604	37/22.8		482	41/25.2		122	23/13.5	
	≥80%	521	90/62.4		486	93/64.2		35	55/37.3	
Site of primary tumour				≤0.001			≤0.001			0.361
	Oropharynx	305	64/41.6		252	72/47.2		53	22/11.8	
	Oral cavity	449	56/42.9		393	58/45.4		56	34/25.2	
	Larynx	215	85/50.9		186	92/56.2		29	37/23.9	
	Hypopharynx	95	31/15.4		81	25/14.9		14	22/14.3	
	Paranasal sinus	43	56/43.4		39	60/45.0		4	23/0.0	
	Nasopharynx	18	29/25.8		17	30/27.5		1	6/0.0	
P16 in Oropharynx-Carcinoma				0.191			0.198			0.329
	Positive	93	72/49.1		75	80/55.7		18	23/0.0	
	Negative	89	54/39.7		75	59/44.8		14	17/10.7	
Grading				0.018			0.188			0.214
	G1	105	64/53.4		99	66/54.2		6	34/50.0	
	G2	657	65/41.9		569	70/45.4		88	27/19.1	
	G3	253	55/37.0		204	61/42.4		49	24/13.4	
T classification				≤0.001			≤0.001			0.064
	T1–2	586	86/54.9		536	89/57.3		50	40/30.0	
	T3–4	539	41/26.9		432	45/30.0		107	27/14.1	
N classification				≤0.001			≤0.001			0.012
	Positive	543	48/29.5		445	53/33.1		98	22/12.9	
	Negative	582	76/52.5		523	83/55.2		59	45/29.1	
M classification				≤0.001			≤0.001			0.182
	Positive	44	9/0.0		34	9/0.0		10	7/12.5	
	Negative	1081	65/42.9		934	71/46.5		147	32/19.9	
UICC stage (8th edition)				≤0.001			≤0.001			0.333
	I–II	451	93/61.4		422	96/63.6		29	34/29.8	
	III–IV	674	45/28.5		546	49/31.2		128	29/16.8	
Treatment received				≤0.001			≤0.001			≤0.001
	Palliative/ BSC	80	6/0.0		28	7/0.0		52	5/4.3	
	Pall. R(C)T	89	12/9.9		69	12/10.2		20	10/10.0	
	Surgery	449	91/62.9		385	99/66.9		64	59/38.3	
	Surgery + adj. R(C)T	184	65/40.6		178	66/40.7		6	31/33.3	
	Def. R(C)T	309	46/32.1		297	48/33.4		12	3/0.0	
	ST	14	10/0.0		11	11/0.0		3	5/0.0	
Treatment recommendation				≤0.001			≤0.001			≤0.001
	Palliative/ BSC	28	7/0.0		28	7/0.0		-	-	
	Pall. R(C)T	94	10/8.4		69	12/10.2		25	5/5.0	
	Surgery	393	97/65.5		385	99/66.9		8	5/0.0	
	Surgery + adj. R(C)T	246	64/40.4		178	66/40.7		68	58/39.5	
	Def. R(C)T	351	42/28.9		297	48/33.4		54	7/5.7	
	ST	13	9/0.0		11	11/0.0		2	1/0.0	
Adherence to treatment recommendation				≤0.001			-			-
	Adherent	968	69/45.1		-	-		-	-	
	Nonadherent	157	31/19.2		-	-		-	-	
Implementation of therapy				≤0.001			-			≤0.001
	Discontinued	78	13/8.5		-	-		78	13/8.5	
	Rejected	79	46/29.5			-		79	46/29.5	
	Carried out	968	69/45.1		968	69/45.1		-	-	
Intention of therapy				≤0.001			≤0.001			≤0.001
	Curative	860	76/49.8		860	76/49.8		-	-	
	Palliative	183	9/5.1		108	11/5.9		75	7/5.7	
	Curative, discontinued	82	49/32.7		-	-		82	49/32.7	
Multivariate Cox Regression Analysis										
Variable			*n* = 1125		HR		95% CI		*p* Value	
Age of diagnosis					1.401		1.112–1.765		0.004	
		≤75	606							
		≥76	519							
Tobacco exposure					1.376		1.065–1.778		0.014	
		Non-smoker	221							
		Current/ former smoker	420							
Charlson comorbidity index					1.419		1.138–1.769		0.002	
		≤5	753							
		≥6	372							
Karnofsky performance status					0.536		0.424–0.678		≤0.001	
		≤70%	604							
		≥80%	521							
UICC stage (8th edition)					2.040		1.617–2.575		≤0.001	
		I–II	451							
		III–IV	674							
Adherence to treatment recommendation					1.779		1.349–2.345		≤0.001	
		Adherent	968							
		Nonadherent	157							

OS, overall survival; HNSCC, head and neck squamous cell carcinoma; UICC, Union for International Cancer Control; BSC, Best Supportive Care; Pall. R(C)T, palliative radio(chemo)therapy; ST, systemic therapy; Adj. R(C)T, adjuvant radio(chemo)therapy; Def. R(C)T, definitive radio(chemo)therapy. ^1^ Proportion of patients alive after a follow-up period of 60 months. ^2^ The *p*-values for the univariate analysis were determined using the log-rank test.

## Data Availability

The data are presented in Table 1, Table 2, Table 3 and Table 4 and Supplementary Material Tables S1–S4.

## Supplementary Materials

The following are available online at https://www.mdpi.com/article/10.3390/cancers14020423/s1, Table S1: Patient and tumour characteristics of the study population treated with curative intent according to adherence/nonadherence to the tumour board recommendation, Table S2: Cross-table comparing the recommended therapy to the received therapy in patients with a curative recommendation, Table S3: Cross-table comparing the therapy recommended by the tumour board to the implementation of the therapy in patients with a curative recommendation, Table S4: Univariate and multivariate analysis of clinicopathologic variables associated with overall survival of patients with a curative recommendation.
