# Supplementary material for: Predictors for Adherence to Treatment Strategies in Elderly HNSCC Patients
Source: Cancers (Basel). 2022 Jan 14;14(2):423. doi: 10.3390/cancers14020423 (PMC8774262; doi:10.3390/cancers14020423)
Supplement: Supplementary file 1 [file cancers-14-00423-s001.zip › cancers-1536283-supplementary.pdf]

cancers

MDPI

Supplementary Material Tables

Table S1. Patient and tumour characteristics of the study population treated with **curative** intent according to adherence/nonadherence to the tumour board recommendation.

Variable	Total	Adherence	Nonadherence	P value
	**n = 990**	**n = 860**	**n = 130**	
Sex–no. (%)				0.743
Male	673 (68.0)	583 (67.8)	90 (69.2)	
Female	317 (32.0)	277 (32.2)	40 (30.8)	
Age at initial diagnosis of HNSCC, years				0.731
Median (range)	75 (26)	75 (26)	74.5 (24)	
Tobacco exposure–no. (%)				**0.006**
Non-smoker	200 (35.1)	184 (37.3)	16 (21.1)	
Current/former smoker	369 (64.9)	309 (62.7)	60 (78.9)	
Pack years				**0.021**
Median (range)	50 (197)	50 (147)	50 (195)	
Alcohol abuse–no. (%)				**0.001**
No ethanol consumption	397 (70.9)	359 (73.4)	38 (53.5)	
Ethanol consumption	163 (29.1)	130 (26.6)	33 (46.5)	
Additional cancer diagnoses–no. (%)				0.569
Other cancers	334 (33.7)	293 (34.1)	41 (31.5)	
None	656 (66.3)	567 (65.9)	89 (68.5)	
Number of additional cancer diagnoses				0.798
0	656 (66.3)	567 (65.9)	89 (68.5)	
1	260 (26.3)	229 (26.6)	31 (23.8)	
≥2	74 (7.5)	64 (7.4)	10 (7.7)	
Charlson comorbidity index–no. (%)				**≤0.001**
≤5	681 (68.8)	609 (70.8)	72 (55.4)	
≥6	309 (31.2)	251 (29.2)	58 (44.6)	
Karnofsky performance status–no. (%)				**≤0.001**
≤70%	484 (48.9)	387 (45.0)	97 (74.6)	
≥80%	506 (51.1)	473 (55.0)	33 (25.4)	
Death due to cancer				**≤0.001**

Survived	476 (54.0)	444 (57.9)	32 (27.8)	
Non-cancer-associated	93 (9.4)	86 (11.2)	7 (6.1)	
cancer-associated	313 (31.6)	237 (30.9)	76 (66.1)	
HNSCC characteristics				
Site of primary tumour–no. (%)				0.748[1]
Oropharynx	245 (24.7)	209 (24.4)	36 (27.7)	
Oral cavity	409 (41.3)	355 (41.3)	54 (41.5)	
Larynx	203 (20.5)	179 (20.8)	24 (18.5)	
Hypopharynx	79 (8.0)	67 (7.8)	12 (9.2)	
Nasal/paranasal sinuses	39 (3.9)	36 (4.2)	3 (2.3)	
Nasopharynx	15 (1.5)	14 (1.6)	1 (0.8)	
P16 in Oropharynx-Carcinoma–no. (%)				0.517
Positive	81 (52.3)	67 (51.1)	14 (58.3)	
Grading–no. (%)				**0.002**
G1	101 (11.2)	95 (12.1)	6 (5.0)	
G2	583 (64.7)	512 (65.5)	71 (59.7)	
G3	217 (24.1)	175 (22.4)	42 (35.3)	
T classification (T)–no. (%)				**≤0.001**
T1-2	559 (56.5)	513 (59.7)	46 (35.4)	
T3-4	431 (43.5)	347 (40.3)	84 (64.6)	
N classification–no. (%)				**≤0.001**
Positive	441 (44.5)	364 (42.3)	77 (59.2)	
M classification–no. (%)				-
Positive	-	-	-	
UICC stage (8th edition)–no. (%)				**≤0.001**
I-II	440 (44.4)	413 (48.0)	27 (20.8)	
III-IV	550 (55.6)	447 (52.0)	103 (79.2)	
Intention of therapy				**≤0.001**
Curative	860 (86.9)	860 (100)	-	
Palliative	48 (4.8)	-	48 (36.9)	
Curative, discontinued	82 (8.3)	-	82 (63.1)	

HNSCC, head and neck squamous cell carcinoma; UICC, Union for International Cancer Control;
[1] The requirements to perform a chi-square test were not fulfilled.

Table S2. Cross-table comparing the recommended therapy to the received therapy in patients with a **curative** recommendation.

		**Treatment Received**						**Total**
		**BSC**	**Pall. R(C)T**	**ST**	**Surgery**	**Surgery + adj. R(C)T**	**Def. R(C)T**	
Treatment recommendation	Surgery	4 (0.4%)	1 (0.1%)	0 (0.0%)	388 (39.2%)	0 (0.0%)	0 (0.0%)	393 (39.7%)
	Surgery + adj. R(C)T	0 (0.0%)	1 (0.1%)	0 (0.0%)	61 (6.2%)	184 (18.6%)	0 (0.0%)	246 (24.8%)
	Def. R(C)T	28 (2.8%)	13 (1.3%)	1 (0.1%)	0 (0.0%)	0 (0.0%)	309 (31.2%)	351 (35.5%)
Total		449 (45.4%)	32 (3.2%)	1 (0.1%)	15 (1.5%)	309 (31.2%)	184 (18.6%)	990 (100%)

BSC, Best Supportive Care; Pall. R(C)T, palliative radio(chemo)therapy; ST, systemic therapy; Adj. R(C)T, adjuvant radio(chemo)therapy; Def. R(C)T, definitive radio(chemo)therapy.

Table S3. Cross-table comparing the therapy recommended by the tumour board to the implementation of the therapy in patients with a **curative** recommendation.

		**Nonadherent**		**Adherent**	**Total**
		**Discontinued**	**Rejected**	**Carried Out**	
Treatment recommendation	Surgery	3 (0.3%)	5 (0.5%)	385 (38.9%)	393 (39.7%)
	Surgery + adj. R(C)T	24 (2.4%)	44 (4.4%)	178 (18.0%)	246 (24.8%)
	Def. R(C)T	37 (3.7%)	17 (1.7%)	297 (30.0%)	351 (35.5%)
Total		64 (6.5%)	66 (6.7%)	860 (86.9%)	990 (100%)

BSC, Best Supportive Care; Adj. R(C)T, adjuvant radio(chemo)therapy; Def. R(C)T, definitive radio(chemo)therapy.

Table S4. Univariate and multivariate analysis of clinicopathologic variables associated with overall survival of patients with a **curative** recommendation.

Univariate Analysis

**Variable**		**Total**			**Adherence**			**Nonadherence**		
		**n = 990**	**Mean OS (months/%[1])**	***p* Value[2]**	**n = 860**	**Mean OS (months/%[1])**	***P* Value[2]**	**n = 130**	**Mean OS (months/%[1])**	***P* Value[2]**
Sex				0.482			0.564			0.386
	Male	673	71/45.6		583	75/49.2		90	30/22.2	
	Female	317	66/47.5		277	70/51.0		40	39/23.0	
Age at initial diagnosis of HNSCC				**0.001**			**0.001**			0.142
	70-74 years	459	76/50.2		394	83/54.6		65	36/22.5	
	75-79 years	340	62/44.2		306	66/46.9		34	25/21.5	
	80-84 years	119	57/39.0		94	59/41.4		25	41/29.0	
	85-89 years	51	47/43.5		48	50/46.4		3	5/0.0	
	Older than 90 years	21	26/30.7		18	30/37.0		3	5/0.0	
Tobacco exposure				**0.003**			**0.014**			0.771
	Non-smoker	200	74/55.5		184	78/58.0		16	27/31.3	
	Current/former smoker	369	61/40.0		309	67/44.0		60	30/18.4	
Alcohol abuse				**≤0.001**			**≤0.001**			**0.049**
	No ethanol consumption	397	47/55.3		359	76/58.5		38	39/23.4	
	Ethanol consumption	163	72/28.0		130	54/32.8		33	18/9.7	

Additional cancer diagnoses				**0.023**			**0.036**			0.059
	Other cancers	334	75/41.0		293	63/44.6		41	22/15.3	
	None	656	58/49.0		567	83/52.6		89	40/25.4	
Charlson comorbidity index				**≤0.001**			**≤0.001**			**0.018**
	≤5	681	79/52.4		609	84/55.0		72	46/30.3	
	≥6	309	45/32.2		251	51/36.7		58	19/13.1	
Karnofsky performance status				**≤0.001**			**≤0.001**			**0.025**
	≤70%	484	43/27.4		387	47/30.0		97	27/16.6	
	≥80%	506	92/63.9		473	95/65.7		33	55/37.3	
Site of primary tumour				**≤0.001**			**≤0.001**			0.749
	Oropharynx	245	75/49,8		209	82/54.6		36	29/16.8	
	Oral cavity	409	60/46.7		355	63/49.8		54	37/27.1	
	Larynx	203	88/52.7		179	94/57.7		24	31/19.0	
	Hypopharynx	79	35/18.4		67	29/17.8		12	26/16.7	
	Paranasal sinus	39	60/48.1		36	64/48.9		3	26/0.0	
	Nasopharynx	15	32/29.2		14	34/31.3		1	6/0.0	
P16 in Oropharynx-Carcinoma				0.297			0.270			0.475
	Positive	81	79/54.6		67	87/60.9		14	26/0.0	
	Negative	74	62/46.4		64	65/51.0		10	22/15.0	
Grading				0.157			0.662			0.335
	G1	101	66/55.1		95	67/56.0		6	37/50.0	
	G2	583	71/46.4		512	76/49.6		71	32/23.4	
	G3	217	62/42.7		175	69/48.7		42	27/15.7	
T classification				**≤0.001**			**≤0.001**			0.101
	T1-2	559	89/57.0		513	92/59.3		46	43/32.6	
	T3-4	431	48/32.3		347	52/36.0		84	31/16.7	
N classification				**≤0.001**			**≤0.001**			**0.028**

	Positive	441	55/34.6		364	61/38.6		77	25/15.1	
	Negative	549	80/55.5		496	86/58.0		53	50/32.4	
UICC stage (8th edition)				**≤0.001**			**≤0.001**			0.519
	I-II	440	95/62.8		402	97/64.4		27	36/32.0	
	III-IV	550	51/33.4		416	53/35.7		103	33/19.7	
Treatment received				**≤0.001**			**≤0.001**			**≤0.001**
	Palliative/ BSC	32	6/3.3		-	-		32	6/3.3	
	Pall. R(C)T	15	12/13.3		-	-		15	12/13.3	
	Surgery	449	91/62.9		385	99/66.9		64	59/38.8	
	Surgery + adj. R(C)T	184	65/40.6		178	66/40.7		6	31/16.7	
	Def. R(C)T	309	46/32.1		297	48/33.4		12	3/0.0	
	ST	1	5/0.0					1	5/0.0	
Treatment recommendation				**≤0.001**			**≤0.001**			**≤0.001**
	Surgery	393	97/65.5		385	99/66.9		8	5/0.0	
	Surgery + adj. R(C)T	246	64/40.4		178	66/40.7		68	58/39.5	
	Def. R(C)T	351	42/28.9		297	48/33.4		54	7/5.7	
Adherence to treatment recommendation				**≤0.001**			-			-
	Adherent	860	76/49.8		-	-		-	-	
	Nonadherent	130	35/22.3		-	-		-	-	
Implementation of therapy				**≤0.001**			-			**≤0.001**
	Discontinued	64	15/10.2		-	-		64	15/10.2	
	Rejected	66	52/33.7		-	-		66	52/33.7	
	Carried out	860	76/49.8		860	76/49.8		-	-	
Intention of therapy				**≤0.001**						**≤0.001**

	Curative	860	76/49.8	860	76/49.8	-	-
	Palliative	48	8/6.4	-	-	48	8/6.4
	Curative, discontinued	82	49/32.7	-	-	82	49/32.7

Multivariate Cox Regression Analysis

**Variable**		**n = 990**	**HR**	**95%CI**	**P value**
Age of diagnosis			1.382	1.068-1.778	**0.014**
	≤75	544			
	≥76	446			
Tobacco exposure			1.327	1.001-1.759	**0.049**
	Non-smoker	200			
	Current/former smoker	369			
Charlson comorbidity index			1.423	1.113-1.819	**0.005**
	≤5	681			
	≥6	309			
Karnofsky performance status			0.572	0.444-0.738	**≤0.001**
	≤70%	484			
	≥80%	506			
UICC stage (8th edition)			1.897	1.481-2.429	**≤0.001**
	I-II	440			
	III-IV	550			
Adherence to treatment recommendation			1.871	1.378-2.541	**≤0.001**
	Adherent	860			
	Nonadherent	130			

OS, overall survival; HNSCC, head and neck squamous cell carcinoma; UICC, Union for International Cancer Control; BSC, Best Supportive Care; Pall. R(C)T, palliative radio(chemo)therapy; ST, systemic therapy; Adj. R(C)T, adjuvant radio(chemo)therapy; Def. R(C)T, definitive radio(chemo)therapy. [1]Proportion of patients alive after a follow-up period of 60 months. [2] The *p*-values for the univariate analysis were determined using the log-rank test.
